# Association of household secondhand smoke exposure and mortality risk in patients with heart failure

**DOI:** 10.1186/s12872-019-1269-y

**Published:** 2019-12-02

**Authors:** Xin He, Jingjing Zhao, Jiangui He, Yugang Dong, Chen Liu

**Affiliations:** 1 Department of Cardiology, The First Affiliated Hospital of Sun Yat-sen University, Guangzhou, China; 2 NHC Key Laboratory of Assisted Circulation, Sun Yat-sen University, Guangzhou, China

**Keywords:** Heart failure, Secondhand smoke, Mortality

## Abstract

**Background:**

Secondhand smoke (SHS) exposure is a well-established cardiovascular risk factor, yet association between SHS and prognosis of heart failure remains uncertain.

**Method:**

Data were obtained from the US National Health and Nutrition Examination Surveys III from 1988 to 1994. Currently nonsmoking adults with a self-reported history of heart failure were included. Household SHS exposure was assessed by questionnaire. Participants were followed up through December 31, 2011. Cox proportional-hazards models were used to assess the association of household SHS exposure and mortality risk. Potential confounding factors were adjusted.

**Results:**

Of 572 currently nonsmoking patients with heart failure, 88 were exposed to household SHS while 484 were not. There were totally 475 deaths during follow-up. In univariate analysis, household SHS was not associated with mortality risk (hazard ratio [HR]: 0.98, 95% confidence interval [CI]: 0.76–1.26, *p* = 0.864). However, after adjustment for demographic variables, socioeconomic variables and medication, heart failure patients in exposed group had a 43% increase of mortality risk compared with those in unexposed group (HR: 1.43, 95% CI: 1.10–1.86, *p* = 0.007). Analysis with further adjustment for general health status and comorbidities yielded similar result (HR: 1.47, 95% CI: 1.13–1.92, *p* = 0.005).

**Conclusion:**

Household SHS exposure was associated with increased mortality risk in heart failure patients.

## Background

Heart failure is a complex clinical syndrome with diverse etiology. It affects 1–2% adults and ≥ 10% elderly population in developed countries [1–4]. The lifetime risk of developing heart failure is so high as 20% for those older than 40 [5]. Prognosis of heart failure remains poor. Absolute mortality rate within 5 years is approximately 50% [6, 7]. Smoking is associated with higher risk of death in patients with left ventricular dysfunction, and therefore, smoking cessation is recommended in patients with heart failure [8–10].

Secondhand smoke (SHS) is another modifiable risk factor for cardiovascular diseases. Studies have shown that SHS also had significant adverse effect on cardiovascular system [11]. Although a previous study using cross-sectional data showed that SHS exposure was associated with lower quality of life in patients with heart failure [12], the effect of SHS exposure on prognosis of heart failure remains uncertain.

Thus, we hypothesized that heart failure patients exposed to household SHS would have higher risk of all-cause mortality. We investigated the association of household SHS exposure and mortality risk using data of the US National Health and Nutrition Examination Surveys (NHANES) III.

## Methods

### Participants

NHANES III is cross-sectional survey in a nationwide probability sample of non-institutionalized civilian resident population in the United States. It is designed to assess the health and nutritional status of adults and children in the United States. From 1988 to 1994, 33,994 sample participants were interviewed and 30,818 participants were examined. Detailed descriptions of plan and operation of NHANES III is available on the website of National Center for Health Statistics [13]. The study was approved by the National Center for Health Statistics Research Ethics Review Board, and a signed informed consent form was obtained from every participant. Our study included currently nonsmoking adults (≥18 years) with a self-reported history of heart failure from survey participation. Participants with a history of non-skin cancer were excluded because of poor prognosis. There were totally 19,592 adults in NHANES III. Among them, 18,810 denied a history of heart failure, and 28 had no idea about their heart failure history. There were 757 participants with a history of heart failure in NHANES III. Among them, 126 current smokers and 59 participants with a history of non-skin cancer were excluded. Finally, 572 heart failure patients were included in the analysis.

### Household SHS exposure

Data on household SHS exposure were obtained from the Family Characteristics section of the interview. The specific question was “Does anyone who lives here smoke cigarettes?” If the answer was yes, the participant was considered to be exposed to household SHS.

### Outcome

Data on mortality status of included participants were obtained from the 2011 Public-Use Mortality Linked File. The file provided mortality follow-up data from the date of survey participation through December 31, 2011. Identifier data were matched to the National Death Index using a probabilistic matching algorithm. A detailed description of the linkage methodology has been published [14].

### Statistical analysis

The primary analytic goal was to assess the association of household SHS exposure and mortality risk in patients with heart failure.

Continuous variables were expressed as mean ± SD, and categorical variables were expressed as percentage. The differences between groups were tested by t test and Pearson’s chi-squared test, respectively.

We calculated unadjusted rates of death per person-year. Kaplan-Meier survival curves and log-rank test were used to evaluate the mortality risk of SHS exposed and unexposed group. We used univariate Cox proportional-hazards models to assess the association of household SHS exposure and mortality risk. Hazard ratios (HRs) and 95% confidence intervals (CIs) were estimated. Follow-up began at the time of interview and ended on the date of death or December 31, 2011, whichever came first. We then used multivariate models to adjust for potential confounders. Model 1 included age, gender, race, education level, family income, urbanization classification of the county, medication, and NHANES III phase. Model 2 included variables in model 1, self-reported general health status, and comorbidities (hypertension, diabetes, hypercholesterolemia, myocardial infarction, chronic kidney disease, COPD/emphysema). Model 3 included variables in model 2, physical functioning, heart rate, systolic, and diastolic blood pressure. However, analysis of Model 3 was exploratory because physical functioning, blood pressure, and heart rate are probably in the causal pathway between SHS exposure and mortality among patients with heart failure. Subgroup analyses were performed among ex-smokers (those who had smoked at least 100 cigarettes) and never-smokers (those who hadn’t smoked 100 cigarettes). A sensitivity analysis was performed by excluding those who died within 6 months. We imputed missing values of covariates using multiple imputation with a Markov Chain Monte Carlo method. Variables listed in Table 1 were included in the imputation model. The assumption of proportional hazards was confirmed for all variables.

**Table 1:** Baseline characteristics of included participants

	Exposed	Unexposed	p
n	88	484	
Male, %	47.3	47.7	0.943
Age, years	66.1 + 17.1	70.8 + 12.6	0.001
Race, %			0.003
Non-Hispanic White	37.5	49.4	
Non-Hispanic Black	31.8	16.3	
Mexican-American	25.0	31.2	
Other	5.7	3.1	
Region, %			0.162
Northeast	12.5	12.4	
Midwest	14.8	17.6	
South	59.1	47.5	
West	13.6	22.5	
Urbanization Classification^a^, %			0.002
1	55.7	61.8	
0	44.3	38.2	
Education level, %			0.759
Education<12y	65.5	67.2	
Education≥12y	34.5	32.8	
Economic Status, %			
Poverty income ratio < 2	64.5	68.2	0.509
Poverty income ratio ≥ 2	35.5	31.8	
Family income< 20,000 dollars	60.4	73	0.019
Family income≥20,000 dollars	39.6	27	
Military service, %	18.2	18.5	0.941
Household Size, %			
1 person	0	124 (25.6)	< 0.001
2 persons	26 (29.6)	223 (46.1)	
≥ 3 persons	62 (70.5)	137 (28.3)	
Medication, %			
ACEI	17 (19.3)	105 (21.7)	0.617
β-Blocker	6 (6.82)	79 (16.32)	0.021
Diuretic	30 (34.1)	216 (44.6)	0.066
Digitalis	25 (29.4)	144 (29.6)	0.799
Ex-smoker, %	51.0	46.6	0.443
Comorbidities, %			
Hypertension^b^	75.0	74.6	0.935
Diabetes^c^	33.0	35.1	0.694
Hypercholesterolemia^d^	71.6	70.3	0.819
COPD/emphysema^e^	22.7	30.2	0.157
Chronic kidney disease^f^	71.5	76.8	0.309
History of myocardial infarction	39.8	52.2	0.033
Self-reported general health status, %			0.535
Poor	31.2	27.9	
Good or fair	68.8	72.1	
Hospitalization during last year	43.2	38.9	0.445
See doctors > 5 times during last year	45.4	51.4	0.311
Physical functioning: Difficulty in walking 10 steps without rest, %			0.148
Yes	62.7	53.5	
No	37.3	46.5	
Systolic blood pressure, mmHg	142.2 + 24.1	141.7 + 23.7	0.849
Diastolic blood pressure, mmHg	77.3 + 14.3	73.8 + 13.5	0.028
Heart rate, bpm	74.4 + 12.3	72.6 + 12.9	0.234
NHANES III phase, %			0.314
1988–1991	54 (61.4)	269 (55.6)	
1991–1994	34 (38.6)	215 (44.4)	
Mean follow-up, years	9.3 ± 7.1	9.2 ± 6.9	0.920

^a^1: Central or fringe counties of metro areas with a population of more than 1 million; 0: All other areas

^b^BP ≥140/90 mmHg or on antihypertensive medications or self-report

^c^HbA_1c_ ≥ 6.5% or on diabetes medications or self-report

^d^Total serum cholesterol ≥240 mg/dL or on anti-cholesterol medications or self-report

^e^Forced expiratory volume 1/forced vital capacity ratio < 0.7 or self-report

^f^Urine albumin to urine creatinine ratio > 30 mg/g or estimated glomerular filtration rate < 60 mL/min/1.73m^2^

All *P* values are two-tailed, and estimates with P values of less than 0.05 were considered statistically significant. All analyses were performed using STATA 13.

## Results

### Baseline characteristics

Baseline characteristics were summarized in Table 1. Eighty-eight patients were exposed to household SHS, and 484 were not. Exposed patients were younger than the unexposed. Patients with household SHS exposure were more likely to be non-Hispanic Black, have a family income of more than 20,000 dollars, and have a larger household size, while those without exposure were more likely to live in an urbanized area, take beta-blockers, and have a history of myocardial infarction. The mean diastolic blood pressure in exposed group was 3.5 mmHg higher than that in unexposed group. Other baseline characteristics were comparable in both groups.

### All-cause mortality

**Fig. 1:**
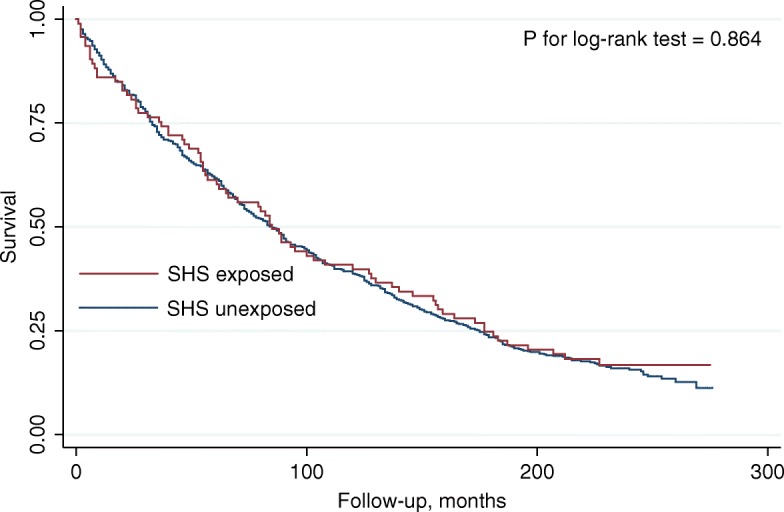
Kaplan-Meier survival curves of household secondhand smoke exposed and unexposed group.

During a mean follow-up period of 9.2 years, there were 475 deaths (72 deaths in exposed group and 403 deaths in unexposed group). Rates of death per person-year were comparable between two groups (88.0 and 90.4 deaths per 1000 person-years in exposed group and unexposed group, respectively) (Table 2). Kaplan-Meier survival curves showed similar results (P for log-rank test = 0.864) (Fig. 1). Results of Cox regression models were summarized in Table 3. In the univariate analysis, household SHS exposure was not associated an increased mortality risk (HR: 0.98, 95% CI: 0.76–1.26, *p* = 0.854). However, after adjusting for demographic variables, socioeconomic variables, and medication in Model 1, household SHS exposure was associated of a 43% increase of mortality risk (HR: 1.43, 95% CI: 1.10–1.86, *p* = 0.007). Model 2 further adjusted for self-reported general health status and baseline comorbidities, and HR for the association was 1.47 (95% CI: 1.13–1.92, *p* = 0.005). Result in Model 3 was consistent. In subgroup analyses, the associations of household SHS and mortality risk did not differ significantly between ex-smokers and never-smokers (p for interaction> 0.1). Excluding death within 6 months did not significantly change the results (HR: 1.38, 95% CI: 1.05–1.82, *p* = 0.020 for Model 1; HR: 1.42, 95% CI: 1.08–1.88, *p* = 0.014 for Model 2; HR: 1.35, 95% CI: 1.02–1.80, *p* = 0.037).

**Table 2:** Mortality incidence rate in household secondhand smoke exposed and unexposed group

	Number of mortality	Person-year	Incidence rate, per 1000 person-year
Exposed	72	817.8	88.0
Unexposed	403	4,48.8	90.4

**Table 3:** Associations of household SHS exposure and mortality risk among currently nonsmoking patients with heart failure

	Univariate Model				Model 1				Model 2				Model 3			
	HR	95% CI	p	p for interaction	HR	95% CI	p	p for interaction	HR	95% CI	p	p for interaction	HR	95% CI	p	p for interaction
Overall	0.98	0.76–1.26	0.864		1.43	1.10–1.86	0.007		1.47	1.13–1.92	0.005		1.40	1.07–1.84	0.016	
Never-smoker	1.02	0.72–1.46	0.889	0.307	1.41	0.96–2.08	0.079	0.639	1.51	1.02–2.23	0.040	0.921	1.39	0.93–2.09	0.112	0.620
Ex-smoker	0.96	0.67–1.37	0.829		1.48	1.02–2.17	0.040		1.46	0.98–2.18	0.066		1.47	0.98–2.22	0.065	

*HR* hazard ratio, *CI* confidence interval

Model 1 adjusted for age, gender, race, education level, family income, urbanization classification, and medications, NHANES III phase

Model 2 adjusted for variables in Model 1, general health status, and comorbidities

Model 3 adjusted for variables in Model 2 and physical functioning, heart rate, systolic, and diastolic blood pressure

## Discussion

This study suggested that household SHS exposure was associated with an increased risk of death among heart failure patients. The results were consistent in analyses with different levels of adjustment. As far as we know, this is the first study to assess the association between household SHS exposure and mortality risk of heart failure.

Suskin et al. analyzed data from the Studies of Left Ventricular Dysfunction trials, and found that active smoking was associated with a 41% increase of mortality risk among patients with left ventricular dysfunction. This study also found no significant difference in mortality risks between ex-smokers and never-smokers, suggesting that smoking cessation could reduce the mortality risk [9]. In our analyses among currently nonsmoking adults, HRs for associations of household SHS exposure and mortality risk ranged from 1.40 to 1.47 with different levels of adjustment. In the subgroup analyses, associations of household SHS and mortality risk were similar among ex-smokers and never-smokers. These results implied that effect of household SHS on mortality risk could be as large as active smoking, and that exposure to household SHS could eliminate the benefit of smoking cessation among patients with heart failure. The substantial effect of household SHS on heart failure prognosis makes it an unignorable risk factor. More attention should be paid on avoiding exposure of household SHS in management of heart failure. Personal smoking cessation will be in vain if a smoke-free environment is not provided.

Unlike multivariate analyses, univariate analyses did not found any significant associations. This could be explained by differences of baseline characteristics, especially age. Among the heart failure patients included in our analyses, those exposed to household SHS were significantly younger, probably reflecting shorter life spans of patients in this group. Harmful effect of household SHS could be covered by survival advantage of younger age. Besides, relatively high family income might also contribute to the differences between univariate and multivariate analyses [15].

The mechanisms of this detrimental effect are still not clear, but there are several possible explanations. Studies have shown that both acute and chronic SHS exposure are associated with decreased heart rate variability, which indicates a shift in the sympathovagal balance toward sympathetic predominance [16–18]. Sustained activation of sympathetic nervous system exerts deleterious effects on cardiovascular system in heart failure patients [19]. SHS exposure also leads to increased inflammation and oxidative stress, which are important in the progression of heart failure [11, 20, 21].

Our study has several limitations. First, history of heart failure was obtained from answer to questionnaire instead of medical record, which could introduce significant recall bias. Second, we lacked information about etiology of heart failure, New York Heart Association class, and ejection fraction, and could not include them in the multivariate analysis. These are important factors that affect the prognosis of heart failure. Third, only a very low proportion of patients in this study took medication for heart failure. Therefore, results of this study needed to be verified in current standard heart failure cohort. Fourth, we did not assess SHS exposure in the workplace, public places, social situations, etc. SHS exposure outside of the household could bias the results. Fifth, the sample size was limited. There were only 88 participants in the exposed group, which might explain the non-significant results in subgroup analyses. Given the limitations mentioned above, results of this study should be interpreted with caution. Sixth, reverse causality could not be ruled out in this study. As family member might pay more attention to patient care if heart failure was more severe. Therefore, advanced heart failure patients might be less likely exposed to household SHS, and the effect of SHS in this study might be underestimated. Seventh, SHS was not included as a time-dependent variable. As SHS exposure status could change during follow-up, using baseline SHS could introduce bias. Eighth, our study assessed SHS only as a binary variable. The dose-response relation of SHS and prognosis of heart failure should be evaluated in detail in future study.

Nevertheless, there were also several strengths in this study. First, using data from a nation-wide survey, the studied participants were representative of heart failure patients in community. Second, current smokers were not included in this study to avoid the influence of active smoking. Although former smokers were included, the effect of former smoking was also evaluated in a subgroup analysis. Third, average follow-up period was almost 10 years in this study, which was long enough to assess the long-term effect of SHS.

## Conclusions

In conclusion, household SHS exposure was associated with increased mortality risk in currently nonsmoking patients with heart failure, which was similar in magnitude to the effect of active smoking. The detrimental effect of household SHS exposure was consistent among ex-smokers never-smokers. Avoidance of household SHS should be a crucial part of management of heart failure.

## Data Availability

Data used in this study were available on the website of National Center for Health Statistics, https://www.cdc.gov/nchs/tutorials/NHANES/Preparing/Download/Frame2_III.htm. The NHANES III database was open to the public and did not require any ethical or administrative permission.

## Abbreviations

CI: Confidence interval
HR: Hazard ratio
NHANES: National Health and nutrition examination surveys
SHS: Secondhand smoke
